# Five novel *EP300* variants expand the genetic and phenotypic spectrum of Rubinstein–Taybi syndrome type 2 in Chinese patients

**DOI:** 10.3389/fgene.2025.1690693

**Published:** 2025-11-20

**Authors:** Qiang Zhang, Qi Yang, Xunzhao Zhou, Zailong Qin, Jingsi Luo

**Affiliations:** 1 Guangxi Key Laboratory of Birth Defects Research and Prevention, Guangxi Key Laboratory of Reproductive Health and Birth Defects Prevention, Maternal and Child Health Hospital of Guangxi Zhuang Autonomous Region, Nanning, China; 2 Department of Genetic and Metabolic Central Laboratory, Maternal and Child Health Hospital of Guangxi Zhuang Autonomous Region, Nanning, China; 3 Department of Child Health Care, Maternal and Child Health Hospital of Guangxi Zhuang Autonomous Region, Nanning, China; 4 Guangxi Clinical Research Center for Pediatric Diseases, Maternal and Child Health Hospital of Guangxi Zhuang Autonomous Region, Nanning, China; 5 Hematology Laboratory, Sheng Jing Hospital of China Medical University, Shenyang, China

**Keywords:** Rubinstein-Taybi syndrome type 2, EP300 gene, whole-exome sequencing, pathogenic variants, genetic diagnosis

## Abstract

**Introduction:**

Rubinstein-Taybi syndrome type 2 (RSTS2; OMIM #613684) is a rare autosomal dominant disorder caused by loss-of-function variants in the *EP300* gene (OMIM #602700), characterized by intellectual disability, distinctive craniofacial features, and skeletal anomalies.

**Methods:**

Whole-exome sequencing (WES) was performed on five pediatric patients presenting with neurodevelopmental delay. Candidate variants were filtered using the TGex platform and validated by Sanger sequencing for familial segregation analysis. The functional impact of variants was assessed using diverse bioinformatic tools, and pathogenicity classifications were assigned according to ACMG/AMP guidelines.

**Results:**

Five novel *EP300* variants were identified in this study: c.4774A>G (p.Lys1592Glu), c.4452 + 5G>C, c.3764A>G (p.His1255Arg), c.3591–2A>G, and c.6439C>T (p.Gln2147*). These alterations impair gene function through mechanisms including amino acid substitution, disruption of mRNA splicing, or premature protein truncation. All variants were classified as pathogenic or likely pathogenic per ACMG/AMP criteria. Literature analysis reveals that the predominant clinical manifestations in the Chinese patients encompassed neurodevelopmental impairment, accompanied by motor delay, growth retardation, and microcephaly. Strikingly, archetypal craniofacial dysmorphisms, such as arched eyebrows, long eyelashes, downslanting palpebral fissures, beaked nose, as well as significant skeletal abnormalities were absent, suggesting *EP300* variants may present with a broader and more variable phenotypic spectrum than previously recognized.

**Conclusion:**

This study reports five novel pathogenic *EP300* variants, expanding the variant repertoire of RSTS2 and providing an important basis for clinical diagnosis and genetic counseling.

## 1 Introduction

Rubinstein–Taybi syndrome (RSTS), also known as broad thumb–hallux syndrome (OMIM #180849), is a rare autosomal dominant neurodevelopmental disorder. It is clinically characterized by intellectual disability, postnatal growth retardation, distinctive craniofacial features (including arched eyebrows, a broad nasal bridge, and downslanting palpebral fissures), and skeletal anomalies, most notably broad thumbs and halluces, along with joint hyperlaxity (Tekendo-Ngongang et al., 2020). RSTS was first described as “broad thumb syndrome” by Michail et al. (1957), and subsequently comprehensively delineated by Rubinstein and Taybi (1963), whose names now designate the syndrome. (Michail et al., 1957; Rubinstein and Taybi, 1963). The estimated incidence ranges from 1 in 125,000 to 1 in 100,000 live births, with approximately 95% of cases resulting from *de novo* variants (Huang et al., 2023). The disorder is primarily caused by pathogenic variants in two genes: *CREBBP* (RSTS type 1; OMIM #600140), which accounts for 50%–70% of cases, and *EP300* (RSTS type 2; OMIM #613684), implicated in 8%–10%. Additionally, approximately 10% of patients harbor microdeletions in the 16p13.3 region (Hennekam, 2006; Hutchinson and Sullivan, 2015).

The *EP300* gene, located at chromosome 22q13.2, encodes the E1A-binding protein p300, a histone acetyltransferase (HAT) (Korzus, 2017). p300 regulates gene expression and maintains chromatin architecture and transcriptional competence through the acetylation of both histone and non-histone substrates (Janknecht, 2002; Tekendo-Ngongang et al., 2020). This multifunctional protein is indispensable for critical cellular processes, including proliferation, differentiation, and stress responses (Garcia-Carpizo et al., 2019; de Thonel et al., 2022; van Voorden et al., 2023). p300 mediates cAMP-responsive gene regulation by being specifically recruited to phosphorylated CREB and also acts as a potent coactivator for hypoxia-inducible factor 1α (HIF-1α), thereby facilitating the expression of hypoxia-responsive target genes such as vascular endothelial growth factor (VEGF) (Zheng et al., 2017).

Comprising 31 exons, the *EP300* gene shares multiple conserved functional domains with its paralog *CREBBP*, most notably the catalytic HAT domain. Consequently, both proteins exhibit substantial functional overlap and participate in the regulation of transcriptional programs, maintenance of cellular differentiation fidelity, modulation of homeostasis, and promotion of developmental processes (Wincent et al., 2016; Chen et al., 2024; Honer et al., 2024).

Pathogenic variants in *EP300* impair p300 function to varying degrees, leading to multisystem developmental anomalies and complex clinical manifestations. For example, variants that disrupt the HAT domain diminish histone H3 acetylation capacity, resulting in transcriptional dysregulation and developmental defects (Lopez-Atalaya et al., 2014; Zhang et al., 2018).

## 2 Materials and methods

### 2.1 Next-generation sequencing

Genomic DNA was extracted from peripheral blood samples of the patients. Target enrichment was performed using the Agilent SureSelect Clinical Research Exome V2 kit (Agilent Technologies, Santa Clara, CA, United States), followed by library construction. Sequencing was carried out on an Illumina HiSeq 2,500 platform (Illumina, San Diego, CA, United States). Raw sequencing reads were aligned to the human reference genome (GRCh37/hg19) using the Burrows-Wheeler Aligner (BWA, v0.7.17). Variant calling and preliminary annotation were performed with the Genome Analysis Toolkit (GATK). Subsequently, variant refinement and prioritization were conducted using TGex software (v5.7, LifeMap Sciences).

### 2.2 Sanger sequencing validation

Candidate variants identified through TGex analysis underwent validation via Sanger sequencing. Primers targeting five variant sites within the *EP300* gene were designed using Oligo7 software (v7.60, Molecular Biology Insights). Primer sequences are detailed in Table 1. Oligonucleotide synthesis was performed by Sangon Biotech (Shanghai, China). PCR amplification proceeded under standard conditions (annealing temperature: 58 °C; 35 cycles), with sequencing performed on an ABI 3730xl DNA Analyzer (Thermo Fisher Scientific).

**TABLE 1:** 5 patients with *EP300* gene variations.

Patient	Variable sites	Status	Parental validation results	Sequencing primer	Clinical interpretation by ACMG/AMP	Significance
Patient-1	c.4774A>G (p.Lys1592Glu)	Heterozygous	*De novo*	5′-TGTGATAAGGAGTGGTAGGG-3′ 5′-CAGGTTTGGAAGGGAATGGA-3′	PS2+PM2_supporting + PP3	Likely pathogenic
Patient-2	c.6439C>T (p.Gln2147*)	Heterozygous	*De novo*	5′-ATTGGGCCAGGTAGGTATCA-3′ 5′-GAGAATTAGGGATCTGCTGG-3′	PVS1+PS2+PM2_supporting	Pathogenic
Patient-3	c.3764A>G (p.His1255Arg)	Heterozygous	*De novo*	5′-GGGGTTGAAGTGAGCTGATA-3′ 5′-TGGGAAGAGAGAAGCTGAGA-3′	PS2+PM2-supporting + PP3	Likely pathogenic
Patient-4	c.4452 + 5G>C	Heterozygous	*De novo*	5′-CCTAGTTTCACCCATCCAAC-3′ 5′-CACACCCCTAAAACCCTAAC-3′	PS2+PM2_supporting + PP3	Likely pathogenic
Patient-5	c.3591–2A>G	Heterozygous	*De novo*	5′-TGTAAGTGGGAAGAGGTGTG-3′ 5′-AAGAGAAGGGTGTAAGGTGG-3′	PVS1+PS2_moderate + PM2_supporting	Pathogenic

### 2.3 Bioinformatic analysis and verification of observations

The functional impact of the prioritized variants was assessed using the following computational tools: CADD (https://cadd.gs.washington.edu/snv), REVEL (https://sites.google.com/site/revelgenomics/), SpliceAI (https://spliceailookup.broadinstitute.org/), MutPred (https://mutpred.mutdb.org/) and RDDC (Rare Disease Diagnostic Center; https://rddc.org). RDDC is a Chinese clinical-grade variant interpretation platform that integrates population frequency, functional prediction, phenotype correlation, and ACMG/AMP classification criteria tailored to East Asian populations. We included RDDC predictions to provide additional evidence from a population-specific resource, thereby enhancing the clinical interpretability of novel variants in our Chinese patients. Protein tertiary structures for *EP300* were generated *in silico* utilizing the SWISS-MODEL server (https://swissmodel.expasy.org/). All variants were classified according to the established ACMG/AMP guidelines (Richards et al., 2015).

## 3 Results

### 3.1 Clinical data

#### 3.1.1 Proband 1

A 2-year-11-month-old male presented with attention deficits and expressive language impairment. He was born at full term via spontaneous vaginal delivery without perinatal asphyxia. Anthropometric measurements were as follows: height 88.4 cm (−1 to −2 SD), weight 11.4 kg (−2 SD), and head circumference 44 cm (<–2 SD). Physical examination revealed impaired social eye contact, lack of response to his name, significant cognitive impairment, and hyperactivity. Cranial MRI showed no structural abnormalities but revealed bilateral maxillary and ethmoid sinus effusions, accompanied by strabismus and torticollis. Gesell Developmental Schedules assessment confirmed global developmental delay affecting adaptive behavior, gross and fine motor skills, and language (Table 2). Biochemical analysis showed elevated levels of total calcium (2.63 mmol/L), iron (6.37 μmol/L), AST (54 U/L), cystatin C (1.34 mg/L), and bicarbonate (21.47 mmol/L), along with reduced glucose (3.79 mmol/L) and prealbumin (163.88 mg/L). No digital anomalies were observed, and cardiopulmonary, hepatorenal, genitourinary, cardiovascular, digestive, and thyroid functions were all within normal limits. WES identified a *de novo* heterozygous missense variant c.4774A>G (p.Lys1592Glu) in exon 29 of the *EP300* gene (NM_001429.3).

**TABLE 2:** Gesell developmental schedules for patient on**e**.

Developmental domains	Developmental age (Months)	Development quotient (DQ)	Level
Adaptability	23.57	73	Mild defects
Gross motor	20.77	65	Mild defects
Fine motor	22.40	70	Mild defects
Language	9.33	29	Severe defects
Sociability	18.43	58	Mild defects

#### 3.1.2 Proband 2

A 3-year-old male presented with speech delay. He was born prematurely at 32 weeks’ gestation (G1P1, non-consanguineous parents) and experienced neonatal hypoxic-ischemic encephalopathy. Birth measurements were: length 50 cm, weight 3.02 kg, and head circumference 34 cm. His clinical history included feeding difficulties with recurrent emesis, frequent respiratory infections, and two afebrile seizures at 6 months of age (interictal EEG was normal). Brain MRI at age 2 revealed periventricular white matter myelination abnormalities. Current anthropometric parameters were: height 95 cm (−1 SD), weight 13 kg (−1 to −2 SD), and head circumference 47.5 cm (−1 to −2 SD). Examination showed attention-deficit/hyperactivity disorder, mild cognitive impairment, and delayed fine motor development, with no other remarkable findings. WES detected a *de novo* heterozygous nonsense variant, c.6439C>T (p.Gln2147*), in exon 31 of *EP300* (NM_001429.3).

#### 3.1.3 Proband 3

A 6-day-old male neonate (G4P2) presented with prenatal polyhydramnios (amniotic fluid index 28.9 cm) and fetal growth restriction (FGR). He was delivered by cesarean section at 37^+2^ weeks due to fetal distress. Birth parameters were: length 44 cm (−3 SD), weight 1.76 kg (−3 SD), and head circumference 29 cm (−3 SD). The immediate postnatal course was complicated by respiratory distress with subcostal retractions and feeding intolerance. Echocardiography confirmed a perimembranous ventricular septal defect. Neurological examination revealed hypotonia and single transverse palmar creases. WES identified a *de novo* heterozygous missense variant, c.3764A>G (p.His1255Arg), in exon 22 of *EP300*.

#### 3.1.4 Proband 4

A 2.5-year-old female presented with motor delay and recurrent respiratory infections. Prenatal diagnosis included right aortic arch, ventricular septal defect, and FGR. Born full-term with no birth asphyxia (birth length 49 cm (−1 to -2SD), weight 2.8 kg (−1 to -2SD). Physical examination demonstrated low hairline, hypertrichosis, and growth retardation (height 86 cm < -1SD; weight 11.7 kg < -1SD, head circumference 46.5 cm < -1SD). Psychomotor delay. Respiratory findings included bronchitis and pertussis (PCR-confirmed). Laboratory studies showed leukocytosis (17.3 × 10^9^/L), glucosuria, and positive *Mycoplasma pneumoniae* IgM. WES revealed a *de novo* heterozygous splice-site variant c.4452 + 5G>C in exon 27 of *EP300*.

#### 3.1.5 Proband 5

**FIGURE 1:**
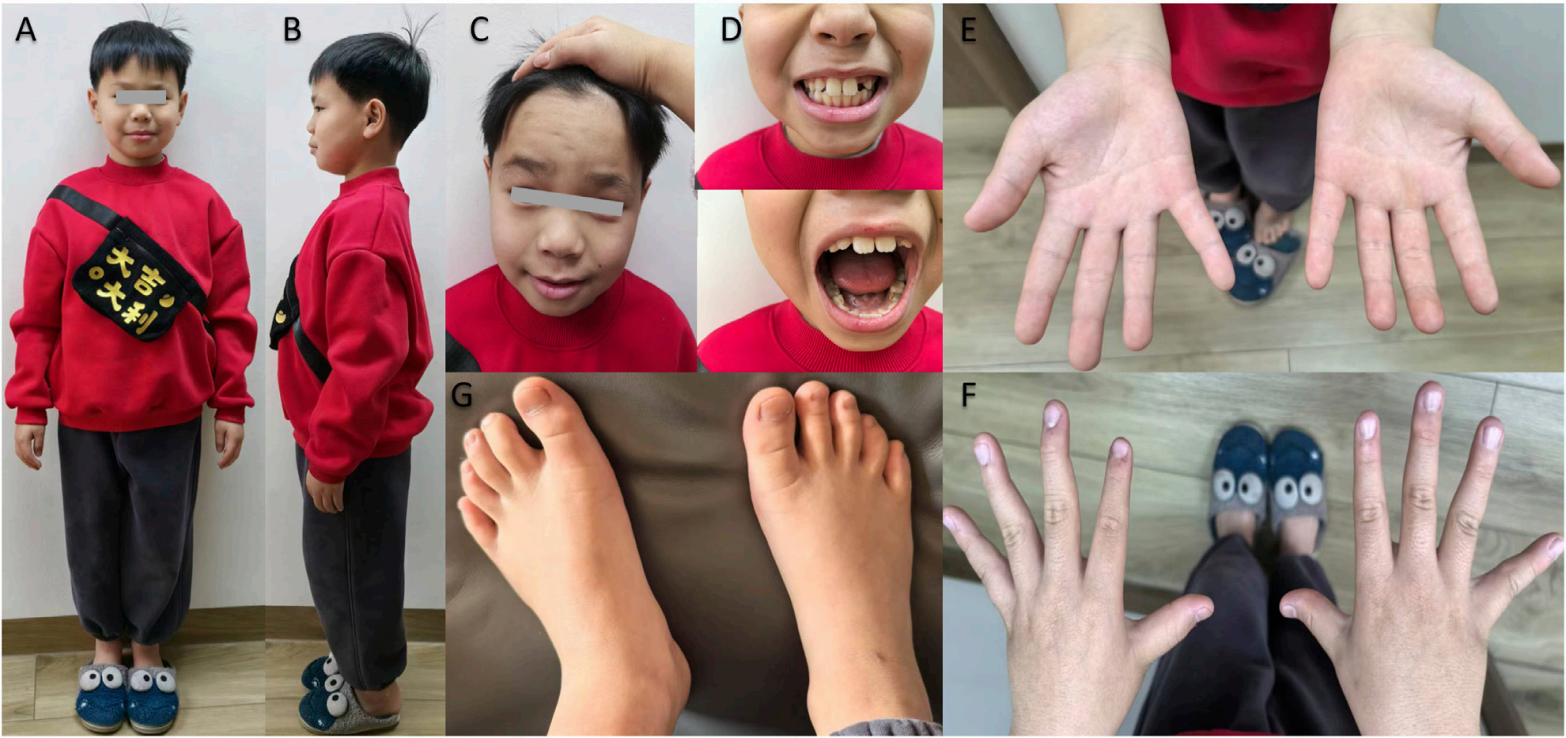
Facial features and distal limb morphology of Proband 5. **(A–D)** Physical examination shows the proband has short stature, microcephaly, and High posterior hairline. **(E–G)** Photographs of the patient’s hands and feet reveal clinodactyly (curved fingers/toes), a broad and flattened hallux.

An 8.5-year-old male presented with intellectual disability and language delay. He was born at 38 weeks’ gestation (G1P1, non-consanguineous parents) following a pregnancy complicated by fetal distress. Birth parameters were: length 50 cm, head circumference 32 cm, and weight 3.0 kg. He exhibited normal development until age 2.5 years and was diagnosed with autism spectrum disorder at age 3. Examination revealed a high posterior hairline, hypertrichosis, and anthropometric deficits (height 126 cm (−1 to −2 SD), weight 24.3 kg (−1 to −2 SD), head circumference 49 cm (<–2 SD)). Notable features included impaired social gaze, attention-deficit/hyperactivity disorder, absence of functional language (limited to involuntary vocalizations), mild intellectual disability, emotional lability, and impaired fine motor coordination. Laryngoscopy confirmed chronic sinusitis with adenotonsillar hypertrophy. Distal limb abnormalities included clinodactyly (curved fingers and toes) and a broad, flattened hallux (Figure 1). WES detected a *de novo* heterozygous splice-site variant c.3591–2A>G in exon 19 of *EP300* (NM_001429.4).

### 3.2 WES and pathogenicity assessment

WES was performed on genomic DNA from the probands, achieving a minimum coverage depth of 20× for over 98% of the target exonic regions. Detailed sequencing metrics for each variant are provided in Table 3. Using TGex software (LifeMap Sciences, United States), we prioritized variants in OMIM-listed genes that were consistent with the patients’ clinical phenotypes. Candidate variants were subsequently validated by Sanger sequencing of PCR-amplified products, with family pedigrees and sequencing chromatograms shown in Supplementary Figure S1. WES identified five novel *EP300* variants (NM_001429.4): two missense variants [c.4774A>G (p.Lys1592Glu) and c.3764A>G (p.His1255Arg)], two splice-site variants (c.4452 + 5G>C and c.3591–2A>G), and one nonsense variant [c.6439C>T (p.Gln2147*)]. In silico analysis using MutPred predicted that the c.4774A>G substitution creates a novel SUMOylation site at lysine 1,590 while disrupting a helical structure, whereas c.3764A>G alters metal-binding capacity. Both modifications are likely to compromise protein function. These missense variants were further supported by multiple computational tools indicating deleterious effects on protein structure and function, fulfilling the ACMG PP3 criterion (Table 4). The nonsense variant c.6439C>T introduces a premature termination codon (PTC) that is predicted to trigger nonsense-mediated mRNA decay (NMD), a conserved eukaryotic quality-control mechanism that degrades PTC-containing transcripts to prevent accumulation of truncated proteins, thereby satisfying the ACMG PVS1 criterion (Udy and Bradley, 2022). Similarly, the splice-site variants c.4452 + 5G>C and c.3591–2A>G are predicted to disrupt canonical splice-site recognition, potentially leading to exon skipping, intron retention, or activation of cryptic splice sites, which could result in frameshifts, premature termination, or structural alterations (ACMG PVS1) (Oh et al., 2024). All five variants were absent in local population controls and in public genomic databases, including gnomAD (v2.1.1; http://gnomad.broadinstitute.org), the 1000 Genomes Project, and the Exome Sequencing Project (ESP6500), supporting the ACMG PM2 criterion. Rare variants are known to exert more pronounced effects on protein integrity and disease susceptibility compared to common polymorphisms (Gorlov et al., 2011). Furthermore, Sanger sequencing of parental samples confirmed that all variants occurred *de novo*, fulfilling the ACMG PS2 criterion (Supplementary Figure S1).

**TABLE 3:** Variant calling Q&R.

Patient	Variable sites	Q&R	READ DEPTH	READ COUNTS (ref,alt)	ALT (%)	GQ	PL	AMP SCORE
Patient-1	c.4774A>G (p.Lys1592Glu)	Med	19	16,3	15.79	68	68,0,0	0.26
Patient-2	c.6439C>T (p.Gln2147*)	High	361	195, 162	44.88	99	3225,0,0	2.87
Patient-3	c.3764A>G (p.His1255Arg)	High	87	47, 40	45.98	99	930,0,0	0.74
Patient-4	c.4452 + 5G>C	High	83	44,39	46.99	99	962,0,0	0.82
Patient-5	c.3591–2A>G	High	119	57,62	52.10	99	1589,0,0	1.18

a&R: Classifies calls by their quality based on GQ, and Coverage, the GQ, mathes genotype quality and coverage matches depth. Low: Coverage <10x and GQ < 15; Med: Coverage <20x and GQ < 50; High: Coverage ≥ 20x and GQ ≥ 50.; Alt: The percentage of reads showing the alternative allele.; GQ: Quality score based on the variant calling.; PL: Genotype likelihood (HOM REF, HET, HOM ALT).; AMP, Score: means Amplification score (coverage/median coverage). In order to check whether the sequencing coverage of variant is abnormally high/low, we use the ratio of variants coverage to median coverage. If AMP, Score >1,the depth of mutant variant is higher than median coverage; if not,the depth is less than or equal to the median coverage. Because of the preference of sequencing, some of the variant depths may be extremely high, so the average value may not be able to reflect the intermediate level of sequencing depth. Therefore, what we choose here is the median sequencing depth instead of average depth.

**TABLE 4:** The impact of the *EP300* variants were predicted using seven *in silico* tools.

Variable sites	CADD	REVEL	Splice-AI	RDDC
c.4774A>G (p.Lys1592Glu)	29.00	0.91	NA	Pathogenic
c.3764A>G (p.His1255Arg)	32.00	0.96	NA	Likely pathogenic
c.4452+5G>C	23.60	NA	0.88 (Moderate, donor gain)	Likely pathogenic
c.3591-2A>G	34.00	NA	0.99 (Strong, acceptor loss)	Likely pathogenic
c.6439C>T (p.Gln2147*)	38.00	NA	NA	Pathogenic

NA:Not Available/Not Applicable.

Based on the ACMG/AMP guidelines, c.4774A>G (p.Lys1592Glu), c.3764A>G (p.His1255Arg), and c.4452 + 5G>C were classified as likely pathogenic, while c.6439C>T (p.Gln2147*) and c.3591–2A>G were classified as pathogenic (Table 1).

### 3.3 Literature review

**FIGURE 2:**
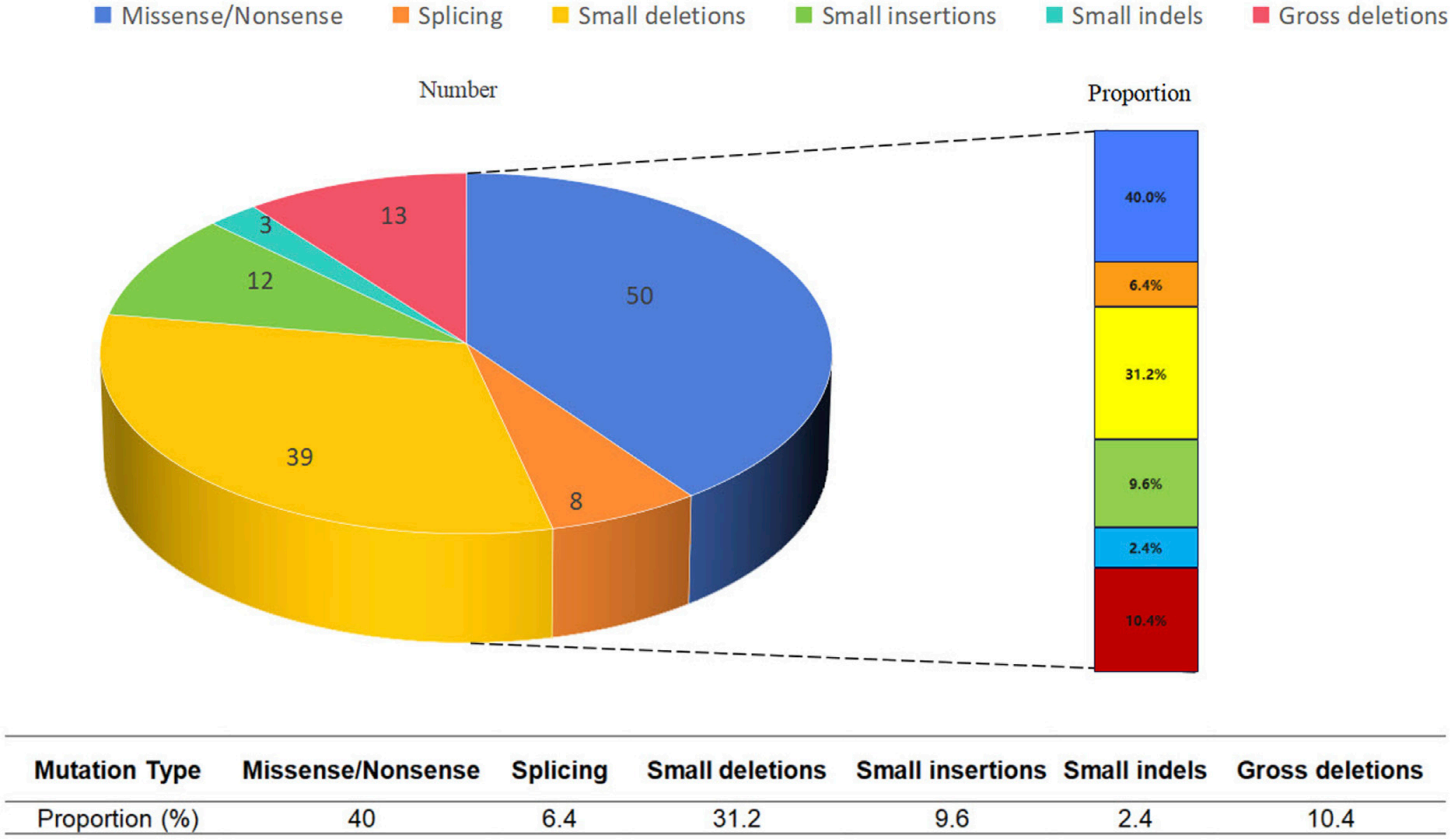
Variant types and their proportions for the EP300 gene based on the HGMD data. Missense/nonsense variants and small deletions are the predominant types of variants.

As of April 2023, the Human Gene Mutation Database (HGMD; https://www.hgmd.org/) documented 125 published *EP300* variants, predominantly missense/nonsense variants and small deletions (Figure 2). To delineate the genetic and phenotypic spectrum of RSTS2 in China, we systematically reviewed literature from OMIM, Wanfang, CNKI, and PubMed databases and compiled all reported Chinese RSTS2 cases (Table 5).

**TABLE 5:** Summary of clinical and molecular features of all patients with *EP300* variants in chinese.

Case ID	P1 (Bai et al., 2023)	P2 (Huang et al., 2023)	P3 (Yang and Chen, 2023)	P4 (Du et al., 2023)	P5 (Du et al., 2023)	P6 (Yang et al., 2024)	P7 (Yang et al., 2024)	P8 (Yang et al., 2024)	P9 (Yang et al., 2024)	P10	P11	P12	P13	P14	Ritio
										Our study					
Gender	Female	Male	Male	Male	Male	Male	Female	Male	Male	Male	Male	Male	Female	Male	Male:Female = 11:3
Age (years)	4	11	9.3	0.7	10.4	5	1.8	11.2	5.9	2.9	3	0.02	2.5	8.5	5.44 ± 3.94
Location (GRCh37)	chr22 41545884	chr22 41558769–41558770	chr22 41556659	chr22 41560078	chr22: 41537062	chr22:41513198–41513201	chr22:41558740	chr22:41573117	chr22 41574921	chr22 41569783	chr22 41574154	chr22 41560092	Chr22 41566580	chr22 41556644	NA
Variant (NM_001429.4)	c.2499dupG (Pro834Alafs*4)	c.3714_3715del (p.Leu1239Glyfs*3)	c.3604G>T (p.Glu1202*)	c.3750C > a (p. Cys1250*)	c.1889A > G (p. Tyr630Cys)	c.104_107delCTCT (p.Ser35Yfs*12)	c.3685G>T (p.Glu1229*)	c.5402G>T (p.Cys1801Phe)	c.7207_7219dup AATTCAAACCTCT (p.Ser2407*)	c.4774A>G (p.Lys1592Glu)	c.6439C>T (p.Gln2147*)	c.3764A>G (p.His1255Arg)	c.4452 + 5G>C	c.3591–2A>G	NA
EXON	14	21	20	22	10	2	21	30	31	29	31	22	27	19	NA
Significance	Pathogenic	Pathogenic	Pathogenic	Pathogenic	Likely pathogenic	Pathogenic	Pathogenic	Likely pathogenic	Uncertain significance	Likely pathogenic	Pathogenic	Likely pathogenic	Likely pathogenic	Pathogenic	NA
Inheritance	*De novo*	*De novo*	*De novo*	*De novo*	*De novo*	*De novo*	*De novo*	*De novo*	*De novo*	*De novo*	*De novo*	*De novo*	*De novo*	*De novo*	100%
Growth retardation	+	-	+	+	+	NA	NA	NA	NA	+	+	+	+	+	9/10 (90%)
Microcephaly	-	-	+	+	-	NA	NA	NA	NA	+	+	+	+	+	7/10 (70%)
Abnormality of the genitourinary system	-	-	+	+	-	NA	NA	NA	NA	-	-	-	-	-	2/10 (20%)
Respiratory problem	+	-	-	+	+	NA	NA	NA	NA	-	+	+	+	-	6/10 (60%)
Psychomotor delay	-	+	+	+	+	+	+	+	+	+	+	-	+	+	12/14 (86%)
Motor delay	+	-	+	-	-	+	+	+	+	+	-	+	+	+	10/14 (71.4%)
Delayed speech and language development	+	+	+	+	+	+	+	+	+	+	+	NA	+	+	13/14 (93%)
Abnormality of the gastrointestinal tract	-	-	+	-	-	NA	NA	NA	NA	-	+	+	+	-	4/10 (40%)
Abnormality of the cardiovascular system	+	-	-	+	-	NA	NA	NA	NA	-	-	+	+	-	4/10 (40%)
Abnormality of finger/toe	+	+	+	-	+	NA	NA	NA	NA	-	-	-	-	+	5/10 (50%)
Abnormal facial features	+	+	+	+	-	NA	NA	NA	NA	-	-	-	+	+	6/10 (60%)
Others	Immunodeficiency; Hypertrichosis Cisternal enlargement	Blurred vision	Hypertrichosis	Hearing impairment Immunodeficiency Hypothyroidism Adrenocortical insufficiency; hyperpigmentation of skin	Immunodeficiency	NA	NA	NA	NA	Hyperactivity Strabismus Torticollis Sinusitis; abnormal biochemical test results	Abnormal myelination Seizures Hyperactivity Premature birth	Premature birth Polyhydramnios Intrauterine growth retardation; Single transverse palmar crease	Intrauterine growth retardation; low anterior hairline; Hypertrichosis; Glycosuria	Sinusitis Enlarged tonsils Increased size of nasopharyngeal adenoids Hyperactivity; Autism	NA

NA:Not Available/Not Applicable.

**FIGURE 3:**
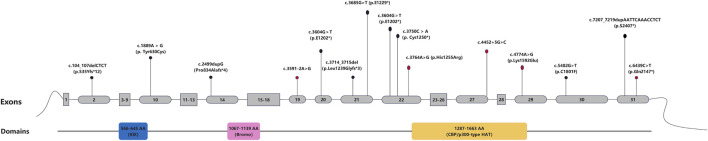
The EP300 pathogenic variants that were reported in China. c.4774A>G (p.Lys1592Glu), c.4452 + 5G>C, c.3764A>G (p.His1255Arg), c.3591–2A>G, and c.6439C>T (p.Gln2147*) are reported for the first time worldwide.

Our study compiled nine previously reported Chinese RSTS2 pedigrees and identified five novel *EP300* variants: c.4774A>G (p.Lys1592Glu), c.4452 + 5G>C, c.3764A>G (p.His1255Arg), c.3591–2A>G, and c.6439C>T (p.Gln2147*), bringing the total number of molecularly confirmed RSTS2 cases in China to fourteen (Figure 3). A comprehensive genotype-phenotype correlation analysis is presented in Table 5 (18–22). In this study’s Chinese RSTS2 case series, all cases were *de novo* variants, with 78.5% (11/14) male and 21.5% (3/14) female, a median age of 5.44 years (range: 6 days–11.8 years). Neurodevelopmental deficits dominated the clinical presentation: language delay (93%), psychomotor delay (86%) and motor impairment (71.4%) were near-universal, accompanied by growth delay (90%) and microcephaly (70%). Additionally, distinctive facial features (60%), thumb/toe malformations (50%), and respiratory issues (60%) were common, and some patients had gastrointestinal/cardiovascular (40%) and genitourinary (20%) abnormalities. In addition, some patients exhibited rare clinical manifestations such as immunodeficiency, visual and hearing impairments, sinusitis, biochemical abnormalities, and seizures. These findings expand the genetic and phenotypic spectrum of RSTS2, offering valuable insights for clinical diagnosis and genetic counseling.

## 4 Discussion

Although *CREBBP* variants constitute the most frequent etiology, the pathogenic role of *EP300* variants in RSTS has gained increasing recognition with advances in genetic testing. *EP300*-associated RSTS (RSTS2) was initially reported in 2007 (Zimmermann et al., 2007), with subsequent cases identified across diverse global populations (Sellars et al., 2016; Al-Qattan et al., 2019; Ismagilova et al., 2025). Current literature indicates fewer than 200 documented RSTS2 cases worldwide, attributed to approximately 100 pathogenic variants (Du et al., 2023; Lacombe et al., 2024).

Multiple studies indicate that patients with *EP300* variants typically exhibit milder phenotypes compared to those with *CREBBP* variants (Korzus, 2017). While RSTS1 and RSTS2 share core phenotypic features, discernible differences exist (Fergelot et al., 2016). Animal models and clinical observations reveal that *EP300*-associated RSTS2 patients demonstrate milder facial dysmorphism (e.g., reduced hypertelorism and less pronounced beaked nose) and lower incidence of broad thumbs/halluces. Instead, they predominantly manifest neurological deficits including anxiety and motor coordination impairments, a pattern corroborated by our case series (Table 5) (Viosca et al., 2010; Fergelot et al., 2016). Additionally, Chinese patients with *EP300* variants frequently present with mild intellectual disability, alongside higher rates of intrauterine growth restriction (IUGR), microcephaly, and prenatal complications. Nevertheless, given the lower prevalence of *EP300* variants, we recommend that targeted testing for *CREBBP* variants should remain the primary approach in the initial evaluation of suspected RSTS cases. Comparative clinical features of RSTS1 and RSTS2 are detailed in Supplementary Table S1.

Notably, *EP300*-associated RSTS2 demonstrates substantial phenotypic overlap with multiple Mendelian disorders, including Floating-Harbor syndrome (FHS; OMIM #136140), Cornelia de Lange syndrome (CDLS; OMIM #122470), and Wiedemann-Steiner syndrome (WDSTS; OMIM #605130) and others. This significant clinical overlap poses diagnostic challenges when relying exclusively on clinical features and conventional ancillary investigations (Huang et al., 2023). Current diagnostic frameworks integrate molecular and clinical criteria for RSTS2, yet genetic testing identifies causative variants in only 55%–70% of clinically suspected cases (Tajir et al., 2013). WES has revolutionized diagnostic precision while concurrently expanding the documented phenotypic spectrum, thereby enabling early and accurate diagnosis. Nevertheless, approximately 30% of individuals with suggestive clinical manifestations lack definitive genetic etiology, potentially attributable to undetected variants in novel genes or regulatory regions (Stevens, 1993).

A comparative analysis of the cases in this study with international cases revealed that Chinese RSTS2 patients share certain clinical features consistent with globally reported cases, including microcephaly, growth retardation, intellectual disability, and delayed speech development (Fergelot et al., 2016). However, the Chinese pediatric patients displayed greater phenotypic heterogeneity, particularly manifesting in rare clinical presentations such as severe, early-onset high myopia (Table 5, Patient 2) and immunodeficiency (Table 5, Patients 1 and 5). Additionally, RSTS2 patients may present with comorbidities including recurrent respiratory infections, gastrointestinal abnormalities (feeding difficulties, gastroesophageal reflux), congenital heart disease, and hypertrichosis (Table 5).

The phenotypic heterogeneity observed in our study may be partially attributed to both the variant class and its location within functionally critical domains of the *EP300* protein. Truncating variants, including nonsense, frameshift, and canonical splice-site alterations, are typically subject to NMD, resulting in haploinsufficiency and a substantial reduction in functional p300 protein levels. In contrast, missense variants lead to single amino acid substitutions and usually produce full-length p300 proteins that escape NMD. The functional consequences of such missense changes are more nuanced and depend on the evolutionary conservation of the substituted residue and its role in the protein’s three-dimensional structure and functional modules; these variants may exert dominant-negative effects or partial loss-of-function.

Notably, the HAT domain catalyzes the acetylation of histone tails, such as H3K27ac, thereby promoting chromatin relaxation and transcriptional activation. This domain represents a critical regulatory hub for chromatin dynamics and gene expression and is highly sensitive to perturbation. Accumulating evidence indicates that missense or inactivating variants within the HAT domain significantly impair *EP300* enzymatic activity and are consistently associated with more severe clinical manifestations, including pronounced growth retardation, classic RSTS features, and multisystem involvement (López et al., 2018).

Conversely, variants located outside the HAT domain, particularly in the C-terminal region including exon 31, often retain partial coactivator function and are associated with milder or atypical clinical presentations. Some scholars classify atypical RSTS phenotypes associated with *EP300* variants as Menke-Hennekam syndrome type 2 (MKHK2) (Menke et al., 2018). A condition characterized by variable intellectual disability and distinct facial dysmorphisms that differ from classic RSTS (Menke et al., 2016). Menke and Hennekam hypothesized that missense variants affecting the terminal region of exon 30 and the initiation region of exon 31 in *CREBBP/EP300* may act through a gain-of-function mechanism to cause MKHK2, whereas variants in other regions typically lead to classic RSTS via haploinsufficiency or disruption of functional domains, most notably the HAT domain (Menke et al., 2018; Ismagilova et al., 2025).

Patients harboring C-terminal variants often present with mild intellectual disability and less pronounced growth delay; some individuals exhibit only subtle dysmorphic features and may be misdiagnosed. Moreover, familial studies have identified carriers of C-terminal variants with minimal or even absent clinical manifestations, suggesting the preservation of residual protein function (Bai et al., 2023).

Our aggregated data from Chinese patients demonstrate that variants in exons 30 or 31 of *EP300* do not invariably result in the MKHK2 phenotype, and conversely, variants outside these exons do not consistently manifest as classic RSTS. Given the limited number of reported cases with *EP300* exon 30/31 variants, further studies are needed to clarify the boundaries between these two syndromic entities.

Although our analysis focused on coding and canonical splice-site variants in *EP300*, non-coding genetic variation may also contribute to the observed phenotypic heterogeneity. Regulatory elements in the 5′/3′untranslated regions (UTRs), promoters, enhancers, or non-coding RNAs could modulate EP300 expression levels, mRNA stability, or splicing efficiency, thereby influencing disease severity or modifying clinical manifestations. In the current bioinformatic pipeline, priority was given to protein-altering variants, and the functional impact of non-coding SNPs was not systematically evaluated. Future studies integrating whole-genome sequencing with comprehensive regulatory annotation are likely to uncover additional genetic modifiers that better explain the variable expressivity of RSTS2.

The genotype–phenotype correlations outlined above provide a plausible mechanistic framework; however, no definitive and consistent association between *EP300* variant type, domain location, and clinical severity has been established, either in our cases or in the broader literature. This uncertainty likely stems from the small number of reported cases and the wide spectrum of clinical features observed across individuals, underscoring the intrinsic phenotypic variability of RSTS2. Nevertheless, integrating molecular characteristics into clinical evaluation remains essential for prognostication and personalized management.

It should also be acknowledged that although the pathogenicity of the variants reported in this study is supported by bioinformatic predictions and ACMG/AMP classification criteria, functional validation experiments, particularly RNA-level validation (e.g., RT-PCR or minigene assays) for the splice-site variants c.4452 + 5G>C and c.3591–2A>G, were not performed due to limitations in funding and access to experimental platforms. This represents a key limitation of our study and underscores the need for further investigation. Future research should prioritize expanding study populations and incorporating both *in vitro* and *in vivo* functional assays to elucidate the biological consequences of these variants and strengthen genotype–phenotype correlations.

## 5 Conclusion

This study identified five novel *EP300* variants (classified as likely pathogenic/pathogenic) in five patients via WES, expanding the variant spectrum of RSTS2 and providing novel evidence for clinical diagnosis and genetic counseling. Compared to the core clinical features of RSTS reported in the literature (intellectual disability, characteristic facies, broad thumbs/halluces, skeletal anomalies, and growth retardation), most patients in this study lacked significant facial dysmorphism, broad thumbs/halluces, or skeletal abnormalities. This highlights the marked phenotypic heterogeneity of RSTS2 and underscores the necessity for comprehensive clinical evaluation. The observed heterogeneity may be closely associated with variant types (e.g., missense, nonsense, splice-site variants exert varying degrees of protein dysfunction) and their locations (variants in critical functional domains such as the HAT domain likely have more severe impacts on protein activity). Despite extensive validation of *EP300*′s central role in RSTS2, definitive genotype-phenotype correlations and pathogenic mechanisms remain unclear, necessitating further exploration of variant functional effects and gene-environment interactions through *in vitro*/*in vivo* models. Current management of RSTS2 focuses on symptomatic and individualized care, including early intervention (e.g., specialized education programs, surgical correction). Future studies should prioritize mechanistic investigations to develop precision therapies for improving patient outcomes and advancing personalized medicine.

## Data Availability

Original contributions presented in the study are included in the article and Supplementary Materials. The raw WES and Sanger sequencing data have been deposited in Figshare (DOI: 10.6084/m9.figshare.29886413).
