# Supplementary material for: Five novel EP300 variants expand the genetic and phenotypic spectrum of Rubinstein–Taybi syndrome type 2 in Chinese patients
Source: Front Genet. 2025 Nov 20;16:1690693. doi: 10.3389/fgene.2025.1690693 (PMC12674597; doi:10.3389/fgene.2025.1690693)

**Pedigree and Sanger sequencing chromatogram of Proband 1**


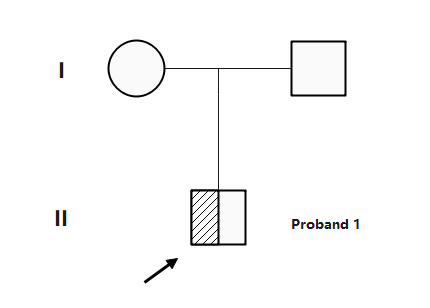


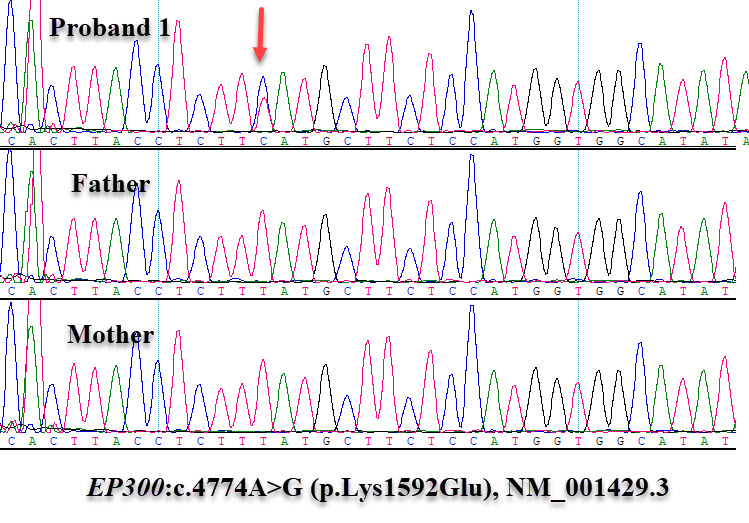


**Pedigree and Sanger sequencing chromatogram of Proband 2**


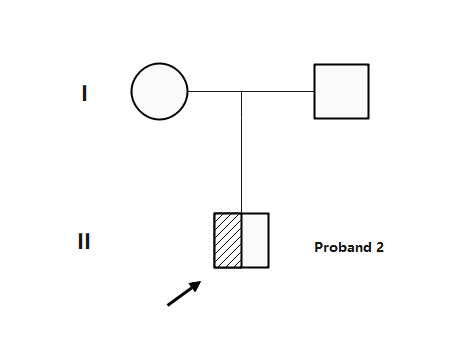


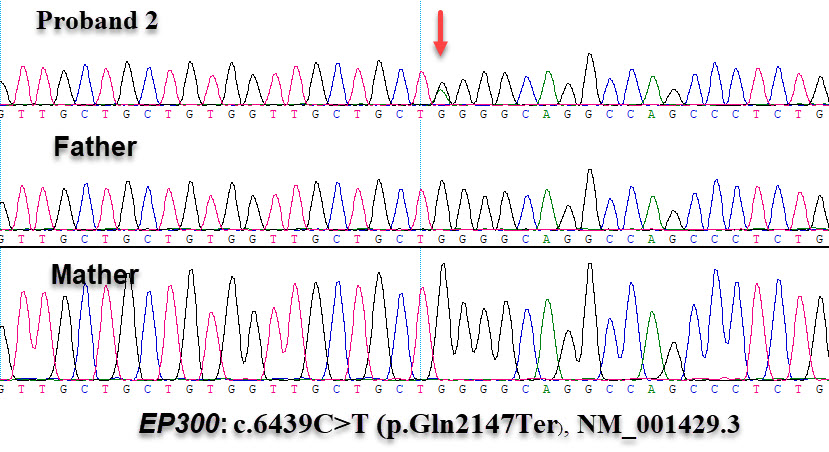


**Pedigree and Sanger sequencing chromatogram of Proband 3**


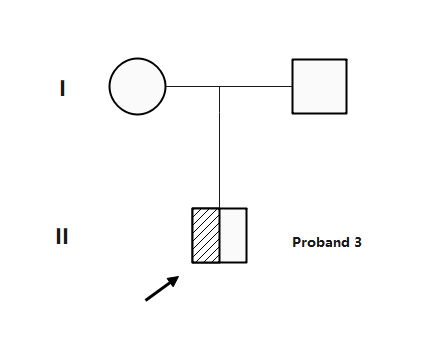


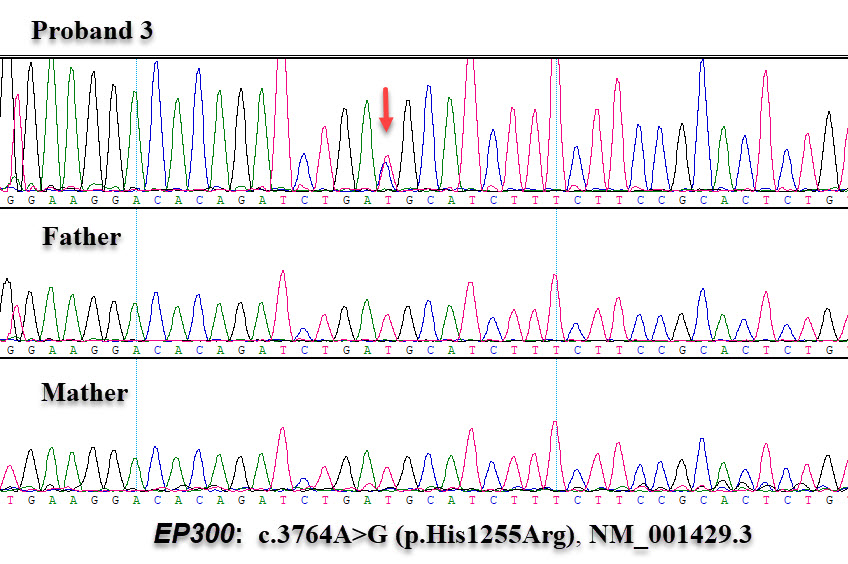


**Pedigree and Sanger sequencing chromatogram of Proband 4**


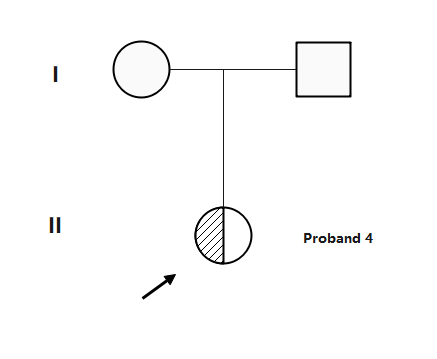


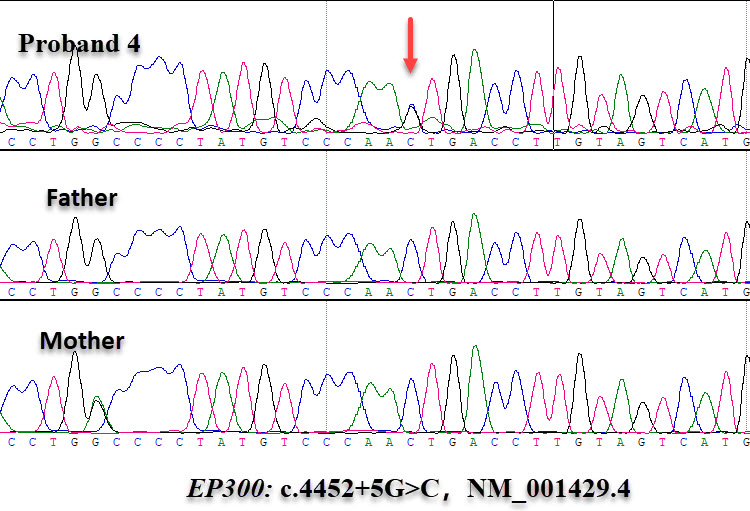


**Pedigree and Sanger sequencing chromatogram of Proband 5**


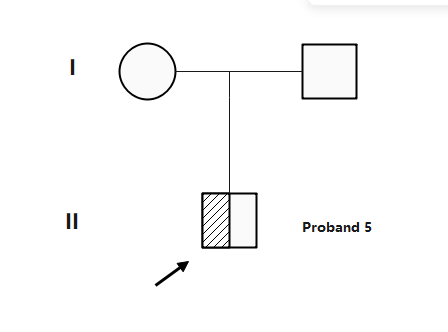


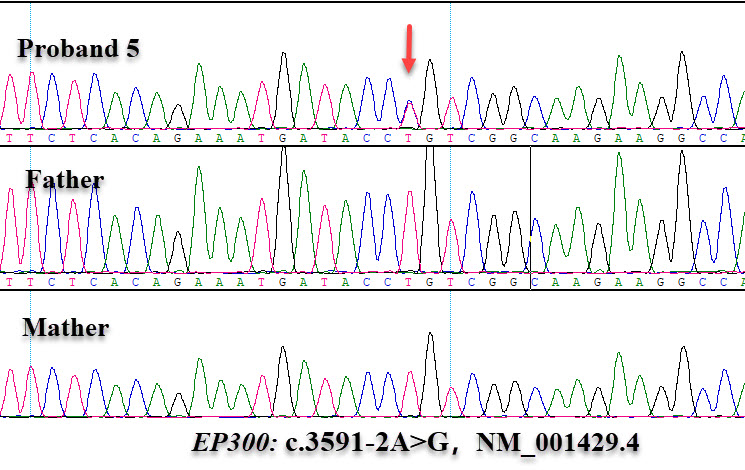

Supplement: Supplementary file 1 [file DataSheet1.doc]
