# Supplementary material for: Five novel EP300 variants expand the genetic and phenotypic spectrum of Rubinstein–Taybi syndrome type 2 in Chinese patients
Source: Front Genet. 2025 Nov 20;16:1690693. doi: 10.3389/fgene.2025.1690693 (PMC12674597; doi:10.3389/fgene.2025.1690693)
Supplement: Supplementary file 2 [file Table1.doc]

| **Supplementary Table 1：Developmental and epileptic encephalopathy (from https://www.omim.org/ )** | | |
| --- | --- | --- |
| **NUMBER** | **#**180849 | **#**613684 |
| **TITLE** | RUBINSTEIN-TAYBI SYNDROME 1; RSTS1 | RUBINSTEIN-TAYBI SYNDROME 2; RSTS2 |
| **GENE** | CREBBP - 600140 | EP300 - 602700 |
| **INHERITANCE**(in 2/2) | - Autosomal dominant | - Autosomal dominant |
| **GROWTH**(in 1/2) | Height  - Short stature - Average adult male height 153 cm - Average adult female height 147 cm |  |
|  | Weight  - Obesity after puberty |  |
|  | Other  - Postnatal growth retardation |  |
| **HEAD & NECK**(in 2/2) | Head  - Microcephaly  - Large anterior fontanel - Late closure of fontanel - Frontal bossing | Head  - Microcephaly |
|  | Face  - Low anterior hairline  - Hypoplastic maxilla - Micrognathia  - Retrognathia  - Grimacing or unusual smile with almost closing of the eyes | Face  - Micrognathia  - Retrognathia |
|  | Ears  - Low-set ears  - Hearing loss - Recurrent otitis | Ears  - Posterior helical pits |
|  | Eyes  - Heavy eyebrows - High-arched eyebrows - Long eyelashes  - Ptosis  - Epicanthal folds - Strabismus - Nasolacrimal duct obstruction - Cataracts - Glaucoma - Coloboma - Downslanting palpebral fissures | Eyes  - Heavy, arched eyebrows - Long eyelashes  - Downslanting palpebral fissures, mild - Normal palpebral fissures |
|  | Nose  - Beaked nose - Deviated nasal septum - Broad nasal bridge | Nose  - Prominent nose  - Beaked nose - Long columella extending below the alae nasi |
|  | Mouth  - Small opening of the mouth - Narrow palate  - High-arched palate | Mouth  - Narrow palate  - High-arched palate |
|  | Teeth  - Dental crowding  - Talon cusps - Crossbite - Screwdriver permanent incisors - Enamel hypoplasia - Enamel discoloration | Teeth  - Dental malocclusion - Overbite - Dental caries |
| **CARDIOVASCULAR**(in 1/2) | Heart  - Atrial septal defects - Ventricular septal defects |  |
|  | Vascular  - Patent ductus arteriosus - Capillary hemangiomas |  |
| **RESPIRATORY**(in 1/2) | - Recurrent respiratory infections |  |
| **CHEST**in 1/2) | Ribs Sternum Clavicles & Scapulae  - Sternal anomalies |  |
| **ABDOMEN**(in 2/2) | Gastrointestinal  - Constipation | Gastrointestinal  - Malrotation (in some patients) - Feeding/swallowing issues beyond the neonatal period (in some patients) |
| **GENITOURINARY**(in 1/2) | External Genitalia (Male)  - Hypospadias - Shawl scrotum |  |
|  | Internal Genitalia (Male)  - Cryptorchidism |  |
| **SKELETAL**(in 2/2) | - Delayed skeletal maturation - Joint hypermobility |  |
|  | Skull  - Large foramen magnum - Parietal foramina |  |
|  | Spine  - Scoliosis - Spina bifida occulta |  |
|  | Pelvis  - Small, flared iliac wings |  |
|  | Limbs  - Patellar dislocation |  |
|  | Hands  - Broad thumbs with radial angulation - Fifth finger clinodactyly - Persistent fetal fingertip pads - Syndactyly  - Polydactyly - Single transverse palmar creases | Hands  - Broad thumbs - Square distal fingertips - Syndactyly (in some patients) |
|  | Feet  - Broad great toes - Plantar crease between first and second toes - Pes planus | Feet  - Broad great toes |
| **SKIN, NAILS, & HAIR**(in 2/2) | Skin  - Single transverse palmar creases - Keloid formation in surgical scars - Capillary hemangiomas - Cafe-au-lait spots |  |
|  | Hair  - Hirsutism | Hair  - Hirsutism (in some patients) |
| **NEUROLOGIC**(in 2/2) | Central Nervous System  - Mental retardation (average IQ 51) - Agenesis of corpus callosum - Severe expressive speech delay - Poor coordination - EEG abnormalities - Seizures - Hypotonia - Hyperreflexia | Central Nervous System  - Mental retardation, mild to moderate - Low-normal intelligence - Autism spectrum disorder (in some patients) - Delayed psychomotor development - Delayed gross motor development - Speech delay - Hypotonia |
|  | Behavioral Psychiatric Manifestations  - Good social contacts - Short attention span - Labile mood | Behavioral Psychiatric Manifestations  - Hyperactivity - Behavioral difficulties |
| **IMMUNOLOGY**(in 1/2) | - Recurrent infections - Polysaccharide antibody response defect |  |
| **NEOPLASIA**(in 1/2) | - Increased risk of tumor formation, especially of the head - Increased risk of leukemia |  |
| **PRENATAL MANIFESTATIONS**(in 1/2) |  | Maternal  - Preeclampsia (in some patients) |
| **LABORATORY ABNORMALITIES**(in 1/2) | - Ten percent of cases are secondary to submicroscopic deletions of 16p13.3 detectable by FISH - A small minority of patients have translocations and inversions involving 16p13.3 |  |
| **MISCELLANEOUS**(in 2/2) | - Incidence of 1 in 100,000 to 125,000 at birth - De novo mutation in most cases - Variable severity - Truncating mutations in CREBBP found in 10% of patients | - De novo mutation - Onset at birth - May have less severe phenotype than RSTS patients with CREBBP mutations |
| **MOLECULAR BASIS**(in 2/2) | - Caused by mutation in the CREB-binding protein gene (CREBBP, 600140.0001) | - Caused by mutation in the 300-KD E1A-binding protein gene (EP300, 602700.0003) |
